# Predictive analytics: beyond the buzz

**DOI:** 10.1186/s13613-019-0524-9

**Published:** 2019-04-11

**Authors:** Frederic Michard, Jean Louis Teboul

**Affiliations:** 1 MiCo, Chemin de Chapallaz 4, Denens, Switzerland; 2 Bicetre University Hospital, Paris South University, Le Kremlin Bicetre, France

Electronic medical records and physiologic monitors produce unprecedented amounts of clinical data, which increasingly powerful computers may turn into novel insights through machine learning and predictive algorithms. Predictive analytics are statistical methods (e.g., random forest models and neural networks) analyzing current and historical data to make predictions about the future. They may detect specific patterns or signatures of clinical deterioration before it becomes overt, opening the door to proactive instead of reactive medicine. As a result, machine learning and predictive analytics, which are subfields of artificial intelligence, are making the buzz in medical journals, congresses, and on the web. In this article, we tried to stay away from novelty blindness to provide a brief evidence-based and balanced overview of opportunities and pitfalls in acute care medicine.

## What can we predict?

First, we can improve the prediction of mortality in patients admitted in the intensive care unit (ICU). Using the super ICU learner algorithm (SICULA), Pirrachio et al. [1] better predicted mortality (area under receiver operating characteristic curve, AUROC = 0.88) than with SAPS-II (AUROC = 0.83) and APACHE-II (AUROC = 0.82) scores. If the differences between AUROC values were statistically significant, they remained small, raising questions regarding their clinical relevance.

Machine learning systems have also been proposed to predict specific adverse events in the ICU and operating theaters. Several attempts have been made to predict intubation [2], prolonged mechanical ventilation and tracheostomy [3], hemorrhage [2], central line-associated bloodstream infections [4], sepsis [2, 5], hypotension [6, 7], and pressure injury [8]. Studies have yielded conflicting results with sensitivities and specificities within the 65–85% range [2–8]. If this level of prediction is definitely better than a random guess, it may not be high enough to make therapeutic decisions in the individual patient. In addition, it remains unknown whether such levels of prediction would result in effective prevention and better clinical outcomes.

Machine learning may help as well on hospital wards. Indeed, predictive algorithms are useful for risk stratification, to detect clinical deterioration at an early stage and to predict ICU readmission [9, 10]. The early recognition of deteriorating patients has been shown to be associated with a decrease in the number of rapid response team calls and ICU transfers [11].

## From predictive to prescriptive analytics

Automation has reduced variability and human errors in non-medical fields. However, closed-loop treatment automation is difficult to implement in clinical practice. Before relying on automated systems, we have to ensure they are going to select the right therapy at the right time. For example, although the automatic titration of vasopressors to ensure a stable blood pressure during surgery is technically feasible, it does not mean that vasopressors are always the right therapeutic answer to a decrease in blood pressure. Depending on the hypotension mechanism, it may actually be wiser to give fluid, or red blood cells, or inotropes, or simply to decrease the depth of anesthesia [12]. In the ICU, the situation is even more complex, since both the interpretation of hemodynamic profiles and therapeutic decisions depend on intricated factors such as patient medical history (cardio-respiratory comorbidities), imaging (echocardiography findings), laboratory tests (lactate), respiratory settings (PEEP level), and treatments received (amount of fluid already administered, dosage of vasopressors). We are not aware of any system, or even prototype, capable of integrating enough information to mimic the human decision-making process in patients with acute circulatory failure.

In contrast, due to its potential to make clinicians life easier with no additional risk for patients, automation of simple diagnostic tests may soon become available. Assessing the hemodynamic impact of respiratory maneuvers (transient rise in tidal volume, end-inspiratory or expiratory pause, lung recruitment maneuver) is known to be useful to predict fluid responsiveness [13]. Patients who do not experience significant changes in hemodynamics during such maneuvers should not receive fluid boluses. These simple tests could be automatized on anesthesia machines and mechanical ventilators so that anesthetists and intensivists would know at regular intervals, and without any additional workload, about the fluid responsiveness status of their patient [14].

## Beyond the peak of inflated expectations

**Fig. 1:**
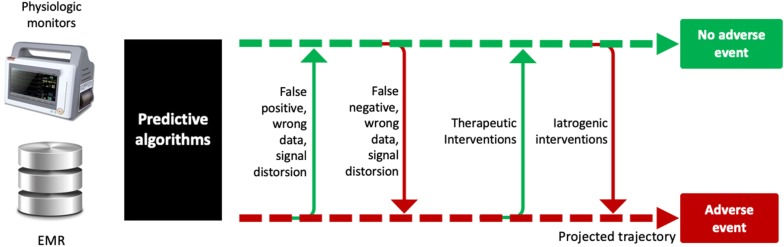
Factors susceptible to affect the predictive value of machine learning algorithms. EMR = electronic medical records. Dotted arrow = projected trajectory, plain arrow = real trajectory

If machine learning and predictive analytics have potential to help us improve quality of care, they are not magic bullets. If you supply the best predictive algorithm with inaccurate information (mistakes are frequent in clinical databases, physiologic signals are often damped or distorted), you will likely end up with wrong predictions (Fig. 1). This is the classical concept of “garbage in, garbage out.” Also, even if machine learning algorithms are able to capture complex, nonlinear relationships in the data, no amount of algorithmic finesse or computer power can squeeze out information that is not present [15]. Finally, predictive algorithms are not crystal balls predicting events before they occur, but rather sniffers detecting that something started to happen—like seismographs telling us that an earthquake is coming before we feel it. Any external intervention, either iatrogenic or therapeutic, is susceptible to change the course of a disease and precipitate, or in contrast prevent, an adverse event (Fig. 1). These interventions are by definition unknown from the predictive system. This may considerably limit the predictive value of machine learning algorithms in acute care settings where multiple interventions per day are usually occurring.

In summary, the concepts of prediction and automation are appealing and, thanks to computer and algorithm innovations, no longer belong to the realm of science fiction. Prediction may lead to prevention and become a useful way to improve quality of care, particularly on hospital wards where patients are monitored less closely than in the ICU. However, several factors may limit the applicability of predictive analytics in real-life conditions. They include the risk to analyze wrong data or distorted signals and the possible interference of external interventions, which are, in essence, unpredictable by a machine. Automation has potential to unload clinicians from repetitive tasks and to prevent human errors. However, the complexity of many therapeutic decisions, and sometimes even the lack of consensus regarding what should be done, is a serious limitation to the implementation of automated systems designed to administer therapy.
